# The perception of fungi among Karajá indigenous children and adolescents from Brazil

**DOI:** 10.1186/s13002-024-00652-5

**Published:** 2024-02-10

**Authors:** Mazulkieliche Jeronimo dos Reis, Lucas Leonardo-Silva, Solange Xavier-Santos

**Affiliations:** Laboratory of Basic and Applied Mycology and Scientific Dissemination (FungiLab), State University of Goiás, Anápolis, Goiás Brazil

**Keywords:** Cerrado, Ethnoknowledge, Ethnomycology, Environmental perception, Mushroom

## Abstract

**Background:**

Environmental perception involves the interpretation and interaction of individuals with their surroundings, influenced by cultural, social, and individual factors. Analyzing the environmental perception of children and adolescents contributes to fostering awareness and ethical behavior toward the environment. Indigenous communities, such as the Karajá from Brazil, possess significant environmental knowledge due to their connection with nature, providing distinctive insights into biodiversity and natural interconnections. In this study, the perception of fungi among Karajá indigenous children and adolescents was investigated.

**Methods:**

The study involved 229 elementary school students from the Macaúba, Fontoura and Santa Isabel do Morro communities, located on Bananal Island, Brazil. Students were encouraged to draw what they knew about fungi and answer where they learned about fungi and what name they give to these organisms. The drawings were analyzed considering seven categories.

**Results:**

The term most used to refer to fungi was *hedoro(u)* (56%), followed by fungus (21%) and mushroom (11%). Most students said they had learned about fungi in nature (38%) and at school (36%). The most represented organisms were in fact fungi (93%), mainly being portrayed in nature (94%). Most participants did not attribute any ecological function to fungi (83%), although 16% of them recognized fungi as decomposers and 1% as phytopathogenic agents. Negative aspects, particularly food contamination, were more frequently represented (13%) than positive aspects (4%). The drawings identified two morphological types: mushrooms (87%) and mold (13%). Among these mushrooms, 68% possibly represent the *Amanita muscaria* species.

**Conclusions:**

Although the children and adolescents showed that they noticed the fungi around them, the group’s concept and understanding were limited to the figure of the mushroom and the negative aspects related to food contamination. Strong association of the fungi with the *A. muscaria* is noteworthy, since it does not occur in the environment in which the participants live, suggesting that external stimuli, such as TV or the internet, can influence their perception more than the nature they are exposed to.

## Background

Fungi are widely distributed in ecosystems and play a key role in maintaining life on Earth, acting as saprobionts, mutualistic symbionts and/or parasites [1, 2]. They have potential use in various industries, such as food, pharmaceuticals and cosmetics, in addition to being useful in environmental recovery, biodegradation and bioremediation [3–5].

Fungi have different forms and can be unicellular (such as yeasts), multicellular (such as molds) and macrofungi (such as mushrooms, bracket fungi, puffballs, among others) [6]. It is estimated that there are 3.8 million fungal species in the world; however, only 120,000 species have been described and accepted [1].

About 6480 species of fungi are known in Brazil, and this diversity is still little explored in the Cerrado [7]. However, studies from the last decade have shown that the biome has a rich diversity [8–10]. Nevertheless, the increasing devastation of the biome, mainly due to agribusiness, has been worrying and has affected biological diversity [11–13]. Although fungi are recognized as a source of natural resources, the lack of studies and shortage of mycologists in the region require intensive research efforts to promote biodiversity and popularize fungi as an ecosystem resource [14]. In this context, it is crucial to raise public awareness of the importance of fungi in the Cerrado to promote the proper conservation of these species.

Environmental perception is the way people perceive and interpret the environment around them. This includes their interactions with natural resources, landscapes and the biodiversity that surrounds them. It can be influenced by cultural, social and individual factors, as well as personal experiences and education. Understanding environmental perception can help promote the conservation and sustainable use of natural resources, as well as understand how environmental changes affect people [15, 16].

In this context, the study of the environmental perception of children and adolescents is an important tool to understand and plan actions that promote awareness and the development of an ethical posture in relation to the environment in which we live. It is critical to continually introduce, discuss, and question children about environmental issues during the early school years. This can foster curiosity, interest and understanding of natural processes and preservation of the environment, in addition to stimulating appreciation of the environment [17–19].

Indigenous peoples have an intimate relationship with nature and consequently a unique and valuable environmental perception. They have deep knowledge of the biodiversity in their territories and understand the interconnection between the different natural elements. The Karajá are an indigenous community from the Brazilian Cerrado that resides on the banks of the Araguaia and Javaés rivers, in the states of Goiás, Tocantins and Mato Grosso. Its villages are located in areas close to the lakes and tributaries of these rivers, as well as in the interior of Bananal Island [20, 21].

The Karajá make up three subgroups, named by themselves according to the position they occupy along the Araguaia: Javaé, Xambioá and Karajá. Each village establishes a specific territory for fishing, hunting and ritual practices, internally demarcating cultural spaces known to the entire group. Considered as belonging to the Macro-Jê linguistic trunk, the Karajá speak the *Iny rybè* language, but also have contact with the Portuguese language [20, 21].

Currently, ethnoknowledge research on the Karajá people primarily focuses on traditional and cultural aspects [21]. However, the knowledge and perception of fungi by the Karajá remain unexplored. The relevance of this research becomes evident when considering the potential repression and loss of traditional knowledge over time. In this context, indigenous children and adolescents play a vital role in the preservation and transmission of this knowledge, as well as in the formation of a solid environmental perception. They are involved in cultural and educational practices, contributing to the preservation of traditions, languages, and connections with the environment, all of which are fundamental to the identity of their communities. Thus, we investigated the perception of children and adolescents of the Karajá ethnic group about Cerrado fungi. We hope to understand how this perception is influenced by traditional knowledge and formal education, and how it will contribute to the conservation and sustainable use of these natural resources in the region.

## Materials and methods

### Study area

**Fig. 1:**
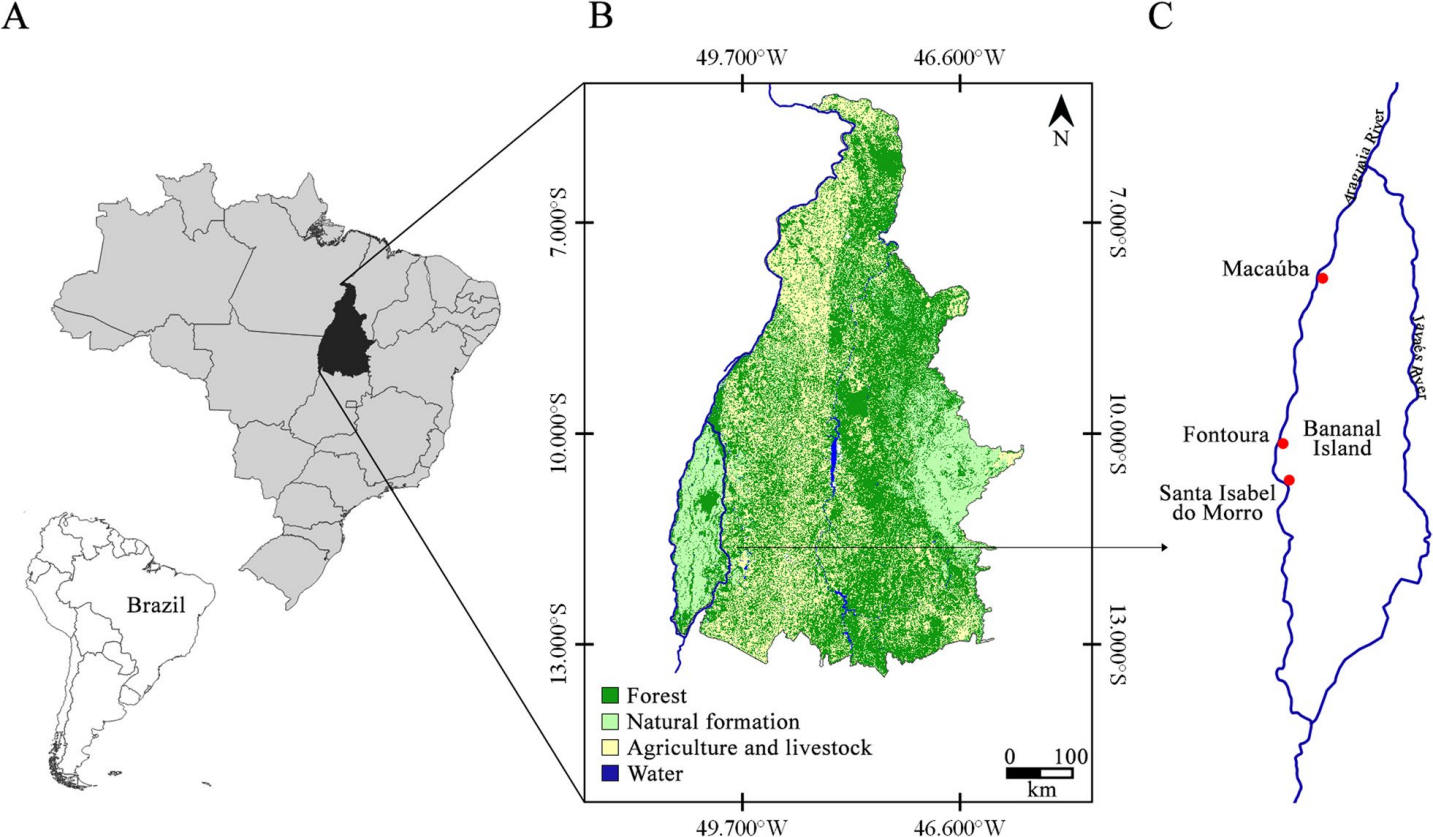
Location of the study area of the Karajá ethnic group. **A** Brazil. **B** Tocantins State [land use and cover (Souza et al. 2020)]. **C** Bananal Island and the dots referring to the Macaúba, Fontoura and Santa Isabel do Morro communities

The Bananal Island is considered the largest river island in the world, possessing an area of 20,000 km^2^ and is bathed by the Araguaia and Javaés rivers. It has been considered an environmental reserve since 1959, and a Biosphere Reserve by UNESCO since 1993. Located in central Brazil in the state of Tocantins, the island is subdivided into the municipalities of Formoso do Araguaia, Lagoa da Conquista and Pium (9° 44′ S and 12° 49′ S, 49° 52′ W and 50° 44′ W). It is located on the border between Tocantins and the states of Mato Grosso (on the Araguaia River) and Goiás (on the southern portion of the Javaés River) [22, 23] (Fig. 1).

**Fig. 2:**
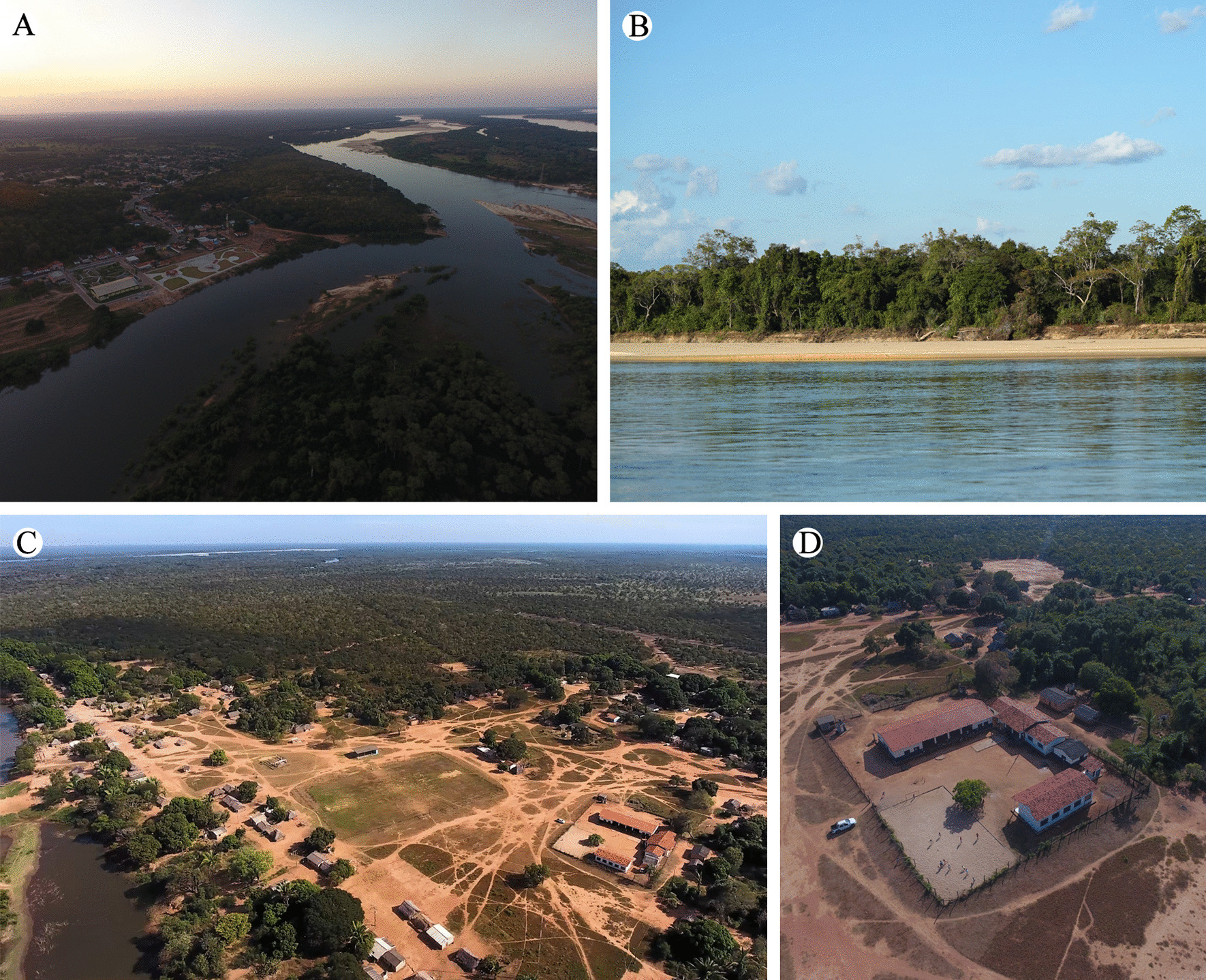
**A** Araguaia River. **B** Landscapes of the Cerrado biome along the Araguaia River. **C, D** Community and school structure

Bananal Island is a transition zone between the Amazon Forest and the Cerrado, characterized as a floodplain area which experiences seasonal flooding (Fig. 2). This area is considered an ecotone, meaning that it is a region of ecological tension between adjacent biomes where there is great genetic diversity [22, 23]. The predominant climate on the island is tropical hot semi-humid AW according to the Köppen climate classification, with a maximum temperature of 38 °C during the dry period (between August and September) and a minimum temperature of 22 °C in July [24].

### Sample universe

Tocantins has a state education network that serves a significant number of indigenous students distributed in several schools. According to the State Secretariat for Education (*Secretaria de Estado da Educação—SEDUC-TO*), around 8056 indigenous students are served in 95 schools which have a total of 549 teachers, with 334 of them being indigenous and with secondary and higher education.

The data collected for this study were obtained from three Karajá indigenous schools: the Maluá Indigenous State School from the Santa Isabel do Morro community, the Heryri Háwã Indigenous State School from the Macaúba community, and the Kumana Indigenous State School, from the Fontoura community. These schools currently serve a total of 973 indigenous students, distributed in elementary school I (1st to 5th year), elementary II (6th to 9th year) and high school (1st to 3rd year), and have 87 professionals, all indigenous, 52 professors and 35 civil servants who work in the administrative sectors. The study involved children and adolescents from elementary school I (4th and 5th grade) and elementary school II (6th to 9th grade), and data collection was carried out in person and, respectively, comprised of the years: 4th (14%), 5th (15%), 6th (12%), 7th (19%), 8th (16%) and 9th (24%).

It should be noted that students have individual rooms for each school year, and the structure of the schools follows the same pattern in each community (Fig. 2). In addition, schools are equipped with internet access and students are allowed to use their mobile devices such as cell phones at specified times and at home. During Elementary School I, there is emphasis on teaching the history and customs of the Karajá culture, as well as the *Iny rybè* native language. Students in Elementary School II have the opportunity to study other subjects, such as Portuguese and Science. The book from the Teláris collection, written by Fernando Gewandsznajder and Helena Pacca, is used for teaching Science.

### Data collection and interpretation of drawings

We used the drawing strategy to analyze the students’ perception of fungi, since drawing is a useful tool to understand how children and adolescents interpret certain subjects. In addition, it is a widely used methodology for studies of environmental perception with children and adolescents [17–19]. The data collection occurred between January and April 2023. We provided students with an A4 sheet of paper, pens and boxes of colored pencils to produce a drawing based on the question: What do you know about fungi? The drawings were only identified with the school year and gender of each student. In addition, two questions were asked on the back of the drawing: (I) Where did you learn about fungi?; (II) What is the name you use to call these organisms?.

A teacher monitored the students during data collection, without interfering with the creative process. There was no time limit for answers and drawings, and students who had difficulties with the language were assisted by translators. We emphasized that the ability to draw was not important, but more the expression of their perception about fungi. No books, images or access to cell phones were allowed during the activity to guarantee impartial perceptions of children and adolescents. These ethical procedures were adopted to guarantee the validity and reliability of the results obtained. After completing the drawings, students had the opportunity to comment (individually) or tell stories related to their works or about fungi in general.

All elements represented by students in their drawings and written responses were entered into a database. The analysis of the drawings was based on the following criteria:

1. The organism(s) that appeared in the drawing (for example, fungus, plant, bacteria, among others);
2. The environment or context in which these organisms were presented (for example, represented in nature, in the domestic or industrial environment, among others);
3. The ecological/functional group (e.g., decomposer, human, animal, and/or plant pathogenic, symbiont, or parasite), based on representation of the fungus on some substrate. We considered the drawings which did not represent the fungi in a specific substrate indeterminate;
4. Characteristics assigned importance (for example, positive or negative). We considered the drawings which only showed the silhouette of the fungi or represented them only as a component of the landscape indeterminate;
5. What role was assigned to the positive and negative aspect (e.g., food rotting, disease causing, or decomposers of other types of organic matter);
6. The morphological type (for example, mildew/mold, mushroom, bracket fungi, jelly fungi, puffball, stinkhorn, among others), following the morphological criteria [6, 25, 26]
7. The organism classified in the drawing at a specific taxonomic level (when possible) or traditional morphological group based on specialized literature [6, 25–28].

Data were processed using Microsoft Excel^©^ program and submitted to descriptive statistical analysis. The frequency between the organisms that appeared in the drawings and the characteristics attributed to importance was compared by association using the chi-squared test. A Correspondence Analysis (CA) was conducted to investigate the relationship between the place of learning about fungi, communities, grade level, age and classification of organisms in the drawings. The GraphPad Prism version 9.0.0 program (GraphPad Software, San Diego, CA, USA, www.graphpad.com) was used to create the percentage graphs, while the CA was calculated with the FActoMineR package [29]. All analyzes were performed using RStudio version 1.2.1335 program [30].

## Results

**Fig. 3:**
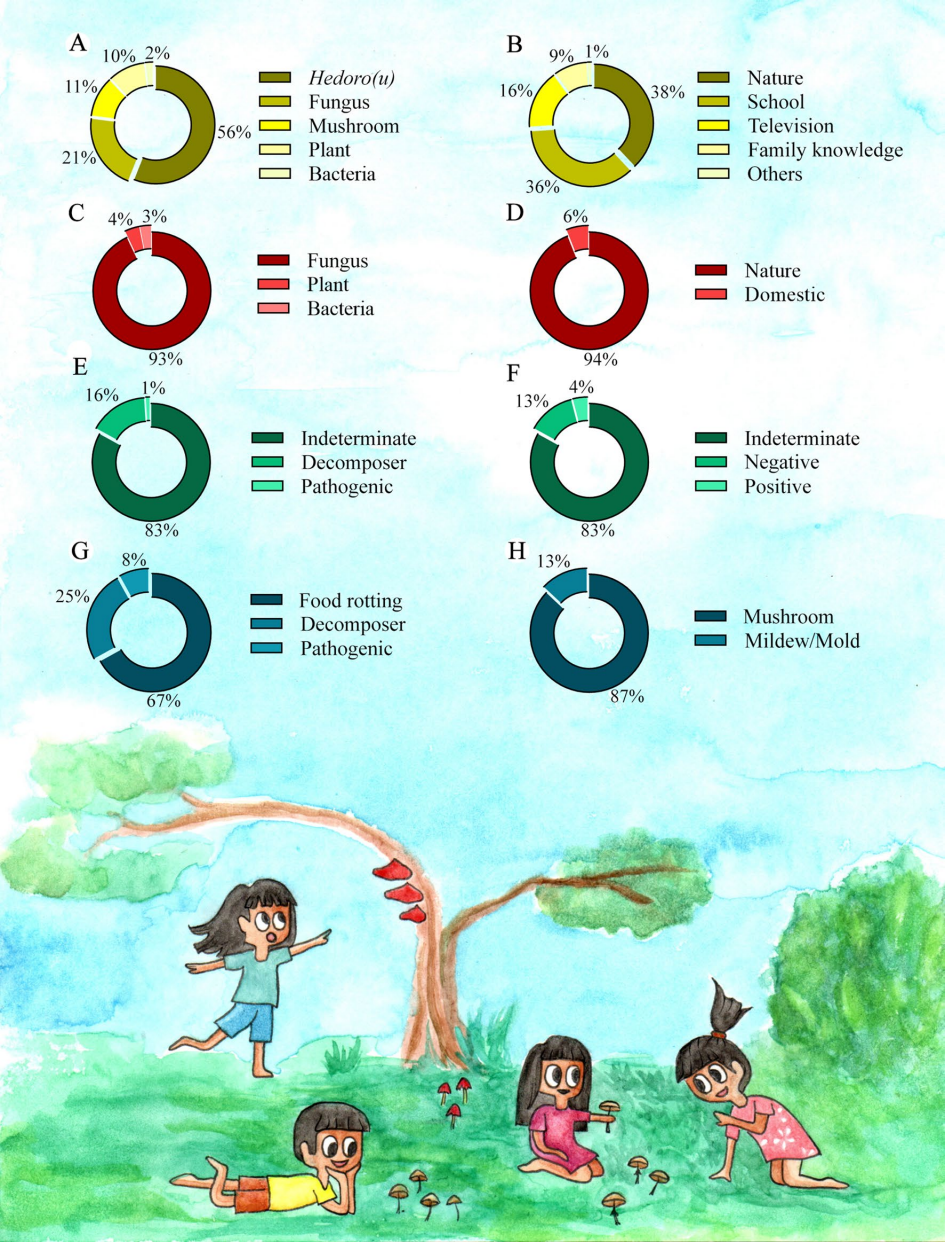
Perception of fungi among indigenous children and adolescents of the Karajá ethnic group from the Fontoura, Macaúba and Santa Isabel do Morro communities. **A** What do you name these organisms? (*n* = 229). **B** Where or from whom did you learn about fungi? (*n* = 229). **C** Organism(s) which appeared in the drawings (*n* = 229). **D** Environment in which fungi were represented (*n* = 213). **E** Ecological/functional group (*n* = 213). **F** Positive or negative importance (*n* = 213). **G** Role attributed to importance (*n* = 36). **H** Morphological type (*n* = 229)

A total of 229 students participated in the study, 103 girls and 125 boys, aged between 8 and 17 years, from the three communities studied (Table 1). According to the children and adolescents interviewed, the term *hedoro*(*u*) is the most used (56%) to refer to fungi (Fig. 3). In addition, their perception of these organisms mainly occurred in nature (38%) and at school (36%).

**Table 1:** Sociodemographic data of indigenous children and adolescents of the Karajá ethnic group, in the Fantoura, Macaúba and Santa Isabel do Morro communities

Community	N	Age range	Sex		Elementary School Year (Grade)					
			Female	Male	4th	5th	6th	7th	8th	9th
Fontoura	70	9‒17	29	41	12	8	7	13	18	12
Macaúba	58	8‒15	34	24	8	10	8	12	12	8
Santa Isabel do Morro	101	8‒17	40	60	11	17	15	18	24	16
Total	229	–	103	125	31	35	30	43	54	36

**Fig. 4:**
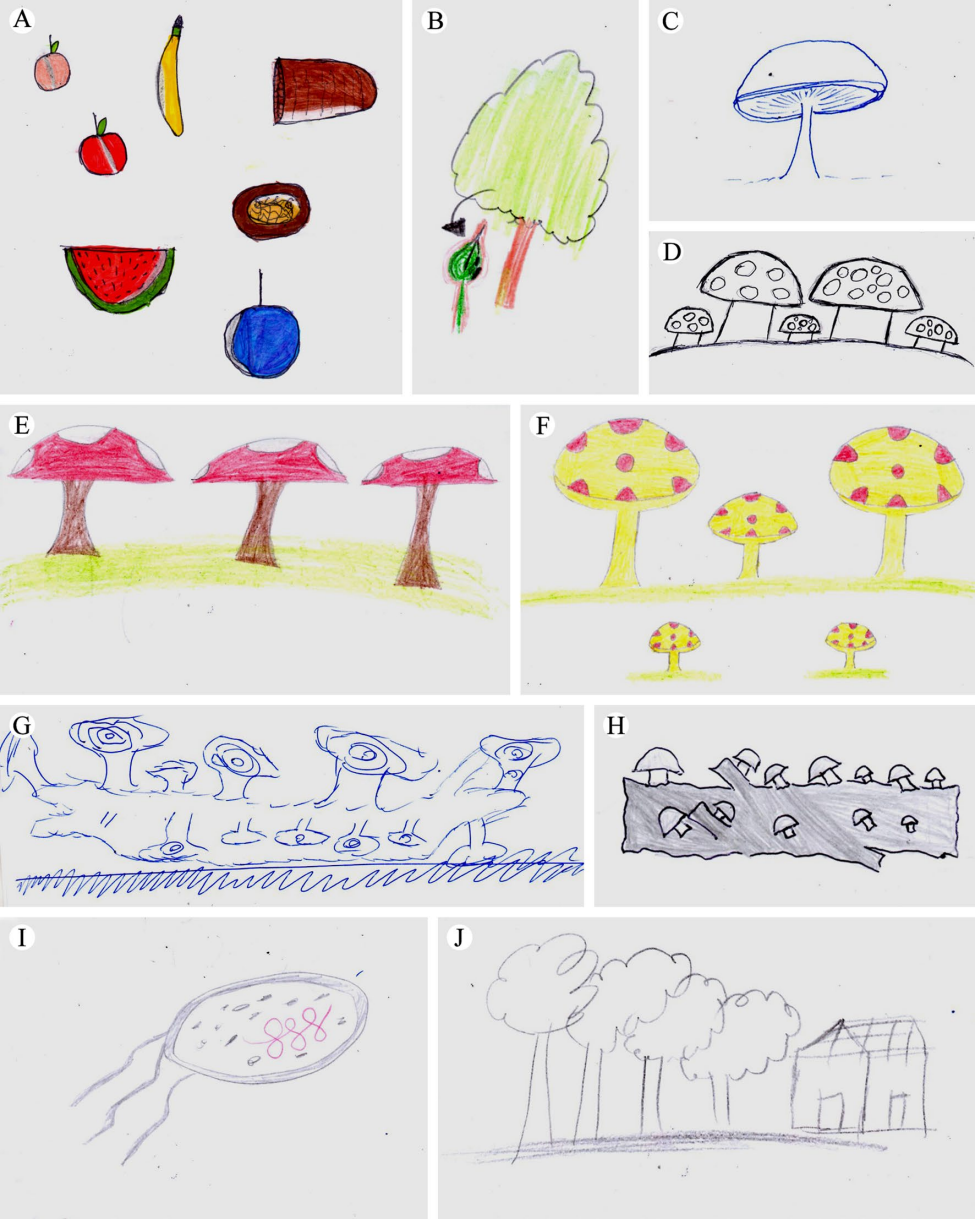
Drawings produced by indigenous children and adolescents of the Karajá ethnic group from the Fontoura, Macaúba and Santa Isabel do Morro communities. **A** Mildew/Mold colonizing food in the domestic environment. **B** Fungi causing plant disease. **C** A mushroom. **D‒F** Probable representation of the *Amanita muscaria* mushroom. **G** and **H** Mushrooms growing on wood. **I** Bacteria. **J** Plant

Fungi were the most represented organisms in the drawings (93%), followed by plants (4%) and bacteria (3%) (Figs. 3, 4). The comparison between the frequency of these drawings showed that children and adolescents know how to differentiate fungi from other organisms (*X*^2^ = 367.06, df = 2, *p* = 0.001). There was no difference in this knowledge between gender (*X*^2^ = 4.6374, df = 1, p = 1) or age (*X*^2^ = 12.602, df = 9, *p* = 0.1815) of the participants. Most drawings represented fungi in nature (94%), while 6% showed their presence in domestic environments (Fig. 3).

The drawings showed that 83% did not attribute an ecological function to fungi, while 16% represented the decomposition of organic matter and 1% portrayed the role of fungi as phytopathogenic agents. However, of those that represented some ecological function, 13% were negative aspects and 4% positive aspects, with a significant difference between the  communities (*X*^2^ = 20.429, df = 4, *p* = 0.0004) and the school year (*X*^2^ = 44.28, df = 10, *p* = 0.002). There was no difference between students’ age (*X*^2^ = 27.973, df = 18, *p* = 0.06). The drawings showed the fungi primarily as food rotters (67%) and wood decomposers (25%) (Fig. 3).

**Fig. 5:**
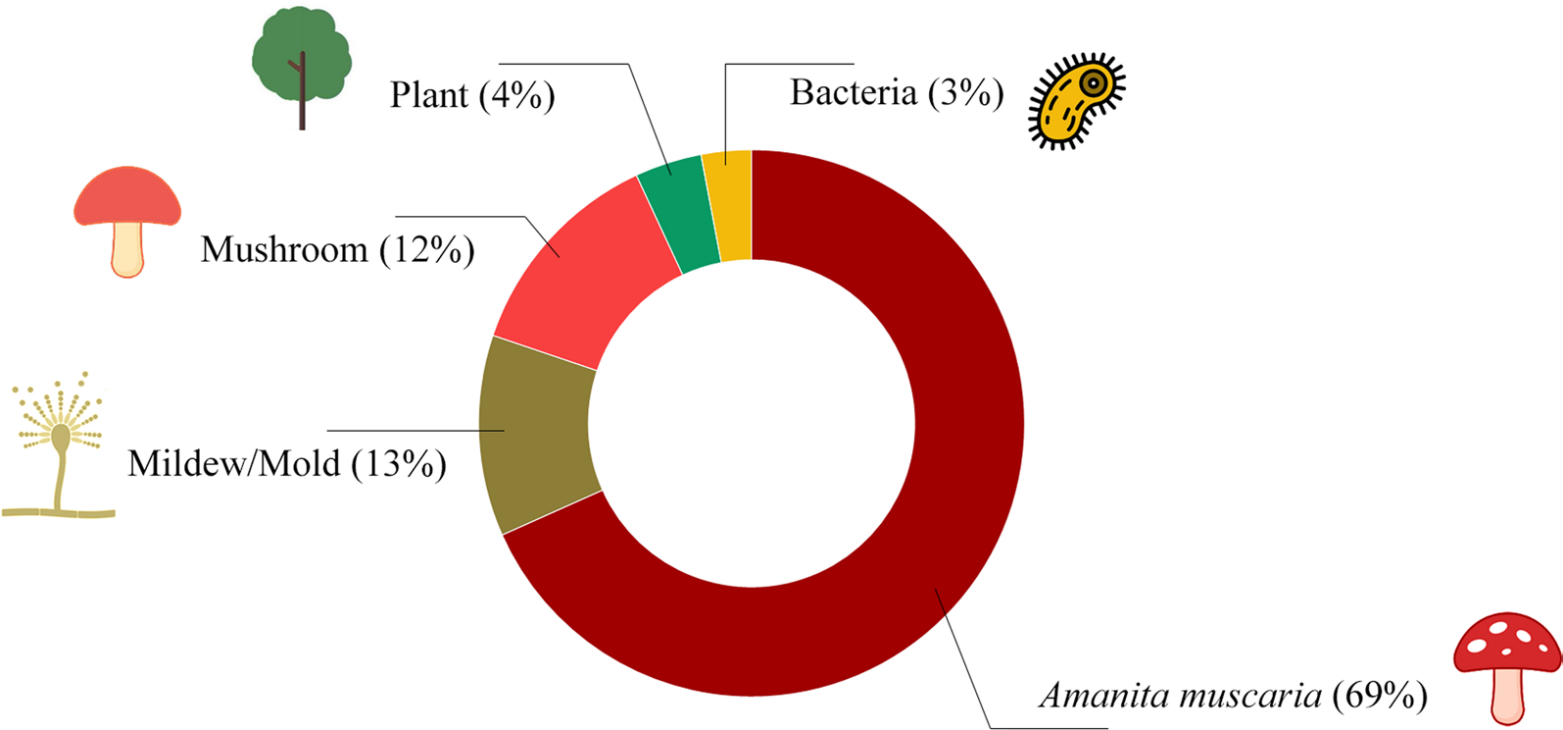
Organisms represented in the drawings of participants from the three communities studied (*n* = 229)

Two morphological types were identified in the drawings: mushroom (87%) and mildew/mold (13%). A total of 68% of the represented mushrooms possibly represent the *Amanita muscaria* species, while 13% and 12% represented other morphological fungi groups (Fig. 5).

**Fig. 6:**
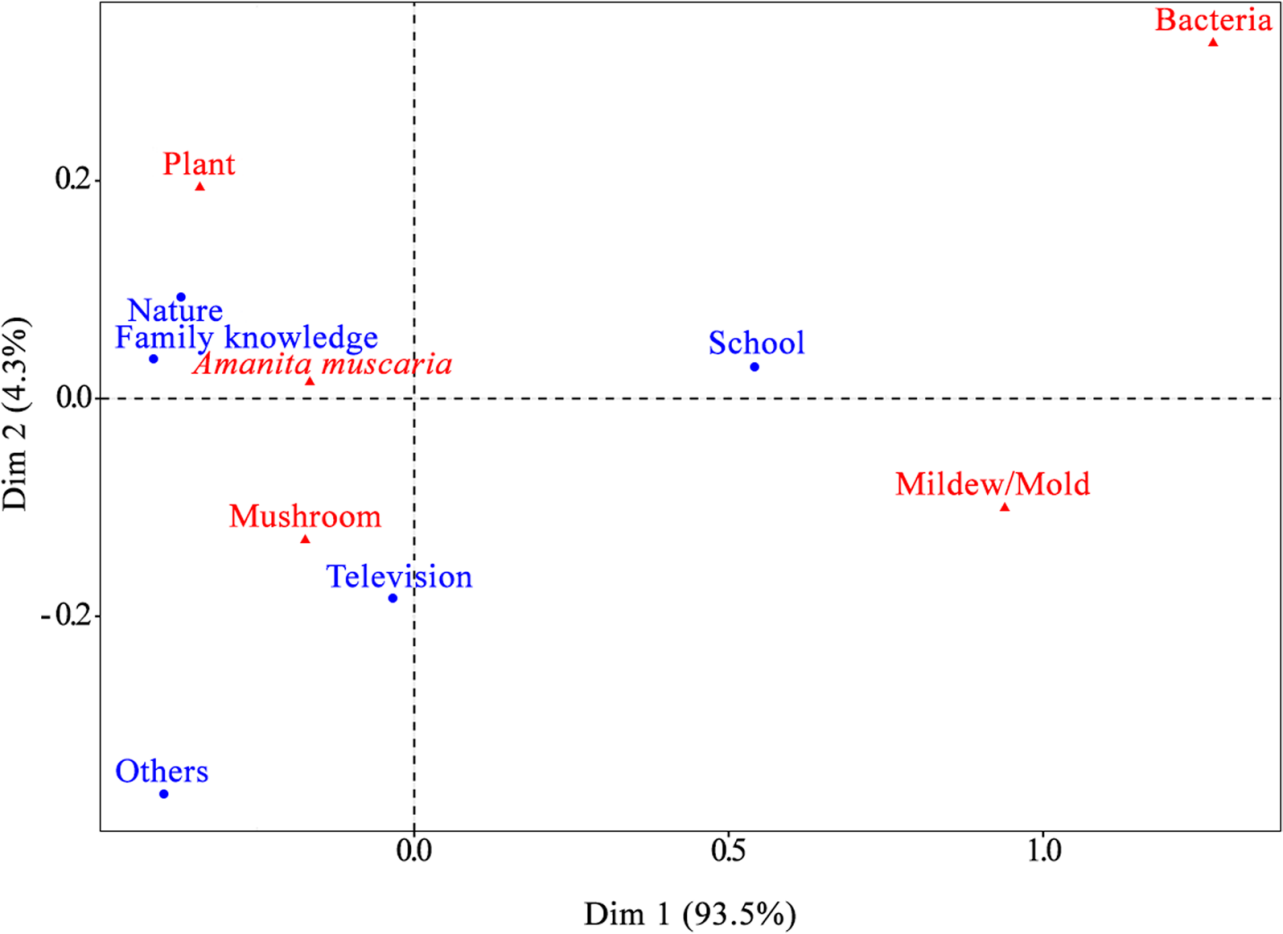
Correspondence Analysis (CA) based on the responses of indigenous children and adolescents of the Karajá ethnic group from the Fontoura, Macaúba and Santa Isabel communities. The terms in blue represent the answers to “Where did you learn about fungi?” and in red the organism classified in the drawings

**Fig. 7:**
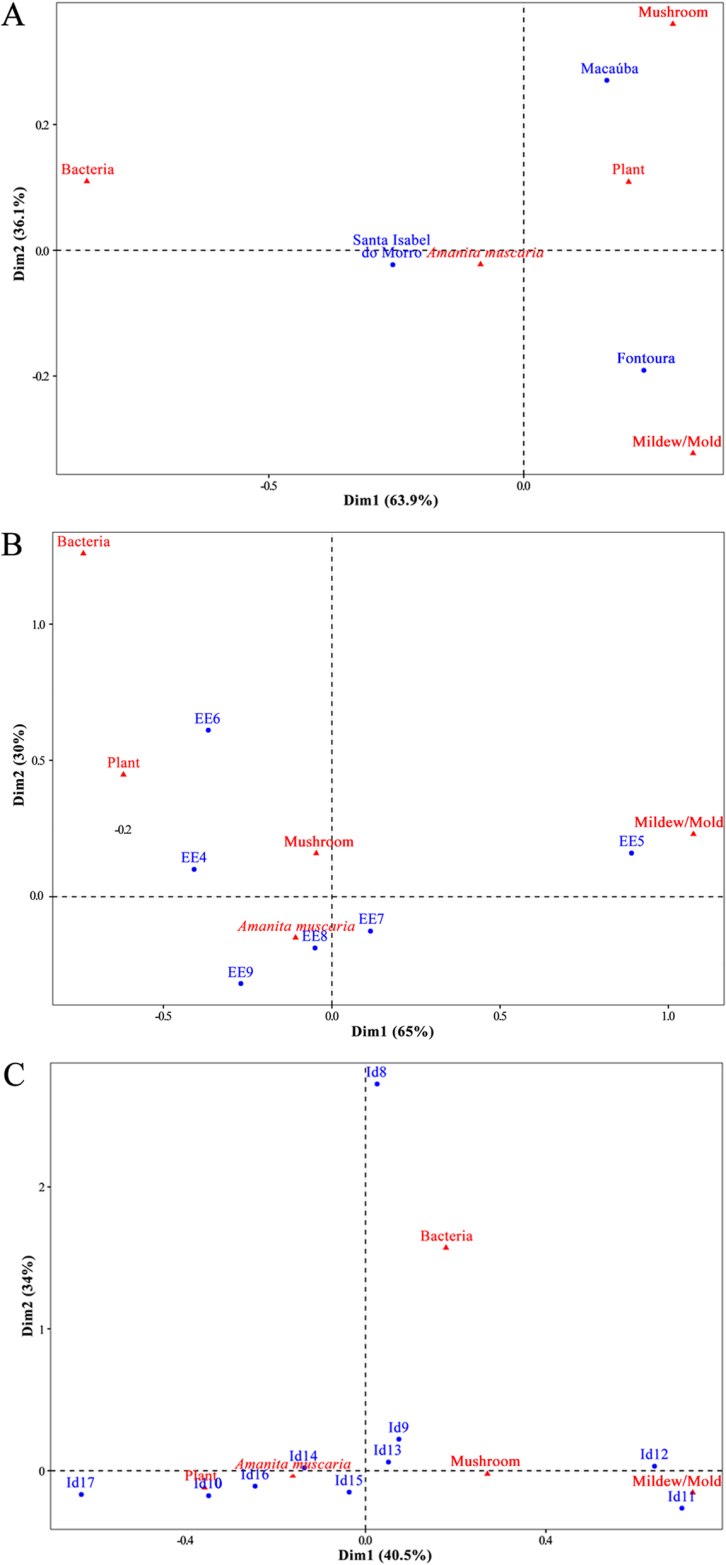
Correspondence Analysis (CA) between communities (**A**), school year (**B**) and age (**C**) and the organism classified in the drawings. The terms in blue represent the communities, school year and age and in red the organism classified in the drawings. EE: Elementary Education; Id: Age

We found a relationship between the organism represented in the drawings and the place where participants learned about fungi (Fig. 6; *X*^2^ = 44.255, df = 16, *p* = 0.0001). It is possible that the fact that mushrooms were more frequently represented in the drawings is due to the influence of television, while the representation of mildew/mold and bacteria is more associated with the school as a place of learning. On the other hand, the perception involving *A. muscaria* and plants mainly occurred among participants who reported nature and family knowledge as a source of learning. The frequency of organisms represented in the drawings differed significantly between communities (*X*^2^ = 18.924, df = 8, *p* = 0.01), school year (*X*^2^ = 62.109, df = 20, *p* = 0.003) and age (*X*^2^ = 53.733, df = 36, *p* = 0.02) of the participants (Fig. 7). Mushrooms were more common in the Macaúba community, while *A. muscaria* was more common in Santa Isabel do Morro. The species was still the most represented in drawings at all ages and school grades, especially between the seventh and ninth grades, and except in the fifth grade, in which mildew/mold was also present.

## Discussion

**Fig. 8:**
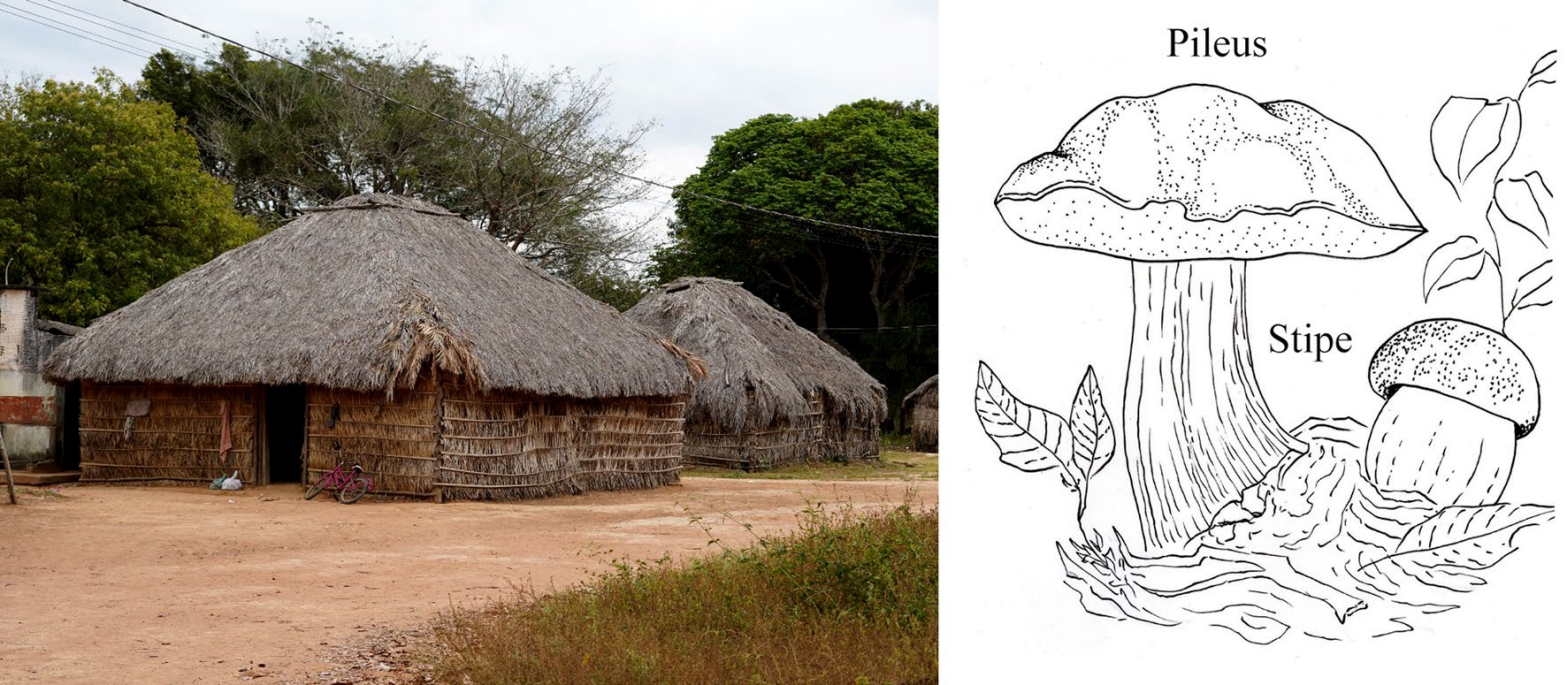
House of residents of the Karajá communities showing the roof of the house, *hedoro*(*u*), similar to the mushroom cap (pileus)

Fungi are mainly called *hedoro*(*u*) in the *Iny rybè* language; the term can be spelled with “oˮ or “uˮ at the end, depending on the gender of the person using it. For example, men should use “uˮ, while women “oˮ. The word means “covering of the houseˮ and refers to the upper, rounded part of the house, which resembles the cap (pileus) of the mushroom (Fig. 8).

The representation of plants and bacteria in the drawings showed that some participants have a misperception about fungi. The conception that fungi are plants is something that is still generally present in society and in many schools and textbooks, despite being a well-known scientific error. The problem is that fungi were historically classified as plants because of their morphological similarities and the lack of knowledge about their biology and unique characteristics. However, today we know that fungi are distinct organisms that belong to a distinct kingdom, the Fungi Kingdom [2].

Although most students included representations of fungi in their drawings, a significant portion did not attribute an ecological function to them. The few drawings that portrayed fungi as decomposing agents or parasites presented them in a negative way, associated with the rotting of food in domestic environments and the emergence of diseases in plants. This negative view of fungi may be the result of a lack of information or a culturally constructed perception. In addition, fungi are often considered just a component of the landscape, as is the case with most of the drawings produced. This can lead to fungi only being associated with negative aspects, such as their ability to cause illness or spoil food. After delivering the drawings, the participants reported that *hedoro*(*u*) is seen as something dangerous in their communities, capable of rotting bread, making the finger of whoever touches it fall off and even causing death. However, they also recognize its cultural use in rituals such as adornments and ornaments, games and in practical activities, such as sanding and finishing ceramics made by older members of the communities.

Despite the participants showing that they perceive the presence of fungi in the environment in which they live, several traditional morphological fungi groups commonly found in the Cerrado, such as poroid (bracket fungi) [9], jelly fungi [8] and gasteroid fungi (puffball, stinkhorn, among others) [10] were not represented in the drawings produced. On the other hand, there was emphasis on mushrooms, indicating that the perception of fungi among most students may be restricted to this morphological group.

Two observed facts could explain this result: the first is that during conversations after data collection in schools, some students and other community members reported that it is common to find some species of mushrooms growing in the straw used to cover houses and in capybara dung (coprophilous fungi). These findings possibly contribute to these people seeing the mushroom species as more familiar. The other fact is related to the possible influence of television media, as well as social networks, which highlight this morphological type to the detriment of other groups of fungi. This relationship was evidenced when we associated the answers about the place where they mentioned that they learned about fungi with the organisms identified in the drawings. We identified that students who illustrated mildew/mold and bacteria indicated the school as a place of learning about these organisms, while students who illustrated the other morphological types of fungi associated this knowledge with nature, family knowledge and television. It should be added that the confusion between fungi and bacteria may probably result from the lack of depth in the content about these microorganisms in the textbook used in schools in the communities, which addressed fungi along with other living beings, making little distinction between them.

The fungal species most represented in the drawings was *Amanita muscaria*. Although the correspondence analysis suggested that this perception is related to the nature and knowledge of the family, it is likely that other factors influenced its high representativeness. For example, the influence of social media may have led participants to represent this species in the wild, even though it does not occur in the Cerrado. This also explains the high frequency of drawings representing the species by students at more advanced ages and school years, who have had access to social media for longer than students in the first years of elementary school. Furthermore, *A. muscaria* is a widely known species and has typical characteristics of mushrooms, which may have led participants to confuse it with similar species found in the region. At this point, it is also relevant to consider the influence of traditional knowledge transmission by the family, as stories about *hedoro*(*u*), which allude to mushrooms, are passed on to younger generations.

*Amanita muscaria* (L.) Lam. (Amanitaceae, Basidiomycota) is a mushroom widely known for its characteristic red hat with white dots and for containing psychoactive compounds such as muscimol and ibotenic acid, which are responsible for the psychoactive effects of this fungus [27, 31]. This species is found in several regions of the world, especially in areas with temperate and boreal climates. There are records of the species in the southern region in Brazil, especially in the states of Paraná, Santa Catarina and Rio Grande do Sul in areas of the Atlantic Forest [27], which possibly result from the introduction of propagules of the fungus associated with *Pinus* sp. seedlings and wood.

Social media has played a significant role in disseminating information on various subjects, including popularization of fungi, especially the *A. muscaria* species. One of the reasons for this is the frequent use of the mushroom’s iconic image in contemporary films, video games, children’s books, art and on various digital platforms such as Instagram, Pinterest, Facebook and YouTube, which attract the attention of the general public, especially younger people. However, the representation of *A. muscaria* in social media usually emphasizes its aesthetic and mysterious aspects, associating it with elements of popular culture such as fairies, elves and fairy tales [32, 33]. This association can generate interest and curiosity, but it can also lead to a mistaken and dangerous understanding of the species, as something harmless and magical. On the other hand, social media are emerging as influential platforms in the transmission of traditional indigenous knowledge [34, 35], which is being lost over time. Studies show that the use of social media by children and adolescents can benefit the preservation and dissemination of local knowledge [36], although vertical and horizontal oral transmission remains predominant in indigenous communities.

Based on the analysis of the graphic and verbal representations of children and adolescents, we noticed the absence of mention or representation of fungi as food, suggesting that this use may not be common among the Karajá. It is important to emphasize that although there are few studies which address the consumption of wild fungi in Brazil, knowledge about the use of these resources as a food source is concentrated in indigenous groups of the Amazon region, especially the Yanomami [37, 38].

The present study proposes strategies to understand the perception of children and adolescents about fungi, while at the same time, identifying the existing gaps in this knowledge in an important indigenous group. It is essential to continuously present, discuss and question environmental issues in elementary school, including fungi, with the aim of arousing curiosity, interest and understanding of the natural processes in which they are involved.

## Conclusion and perspectives

Based on the data obtained, it is observed that although children and adolescents of the Karajá ethnic group perceive the presence of fungi around them, the concept and importance of the group are predominantly restricted to the figure of the mushroom and the negative aspects in the relationship with the humans, such as food spoilage. Although our study is a pioneer in Brazil, the results when verifying the perception of indigenous children and adolescents about fungi through drawings indicate the effectiveness of the strategy in collecting information, enabling better understanding of the relationship of these individuals with the fungi present in their environment. However, it is important to emphasize that the children’s reports about fungi provided details that were not represented in the drawings or that were not noticed.

We verified that learning about fungi begins in childhood, being acquired through personal observations of species present in the thatched roof of houses or in animal feces, such as the capybara; or even through oral transmission by older members of the family, who tend to mainly emphasize the negative cultural aspect of fungi as dangerous or harmful beings from which one should keep their distance. This attitude contributes to the dissemination of legends, myths and superstitions, resulting in a culture in which fungi are little used for different purposes.

This information serves as perspectives and contributes to develop more effective educational strategies about fungi in indigenous schools. It is important to highlight the relevance of learning about these organisms both in nature and at school, which can be achieved through disseminating scientific information about fungi. Thus, it is possible to promote a change in the perception of individuals in relation to fungi and their benefits, contributing to develop more sustainable practices in relation to these organisms.

For future studies, we recommend adopting complementary data collection methods, such as taking guided trails so that children can get to know the local fungi and its importance in a more direct and participatory way. In addition, after completing the study, it is important to return the results to the communities through development of educational products aimed at popularizing knowledge about fungi. This initiative can promote greater involvement of communities with the theme, as well as contributing to appreciate traditional knowledge and the preservation of local biodiversity.

## Data Availability

All data are available in this paper.

## Abbreviations

CA: Correspondence analysis
ICF: Informed consent form
IAT: Informed assent term
